# Occurrence, Distribution, and Management of Aphid-Transmitted Viruses in Cucurbits in Spain

**DOI:** 10.3390/pathogens12030422

**Published:** 2023-03-07

**Authors:** Celia De Moya-Ruiz, Pedro Gómez, Miguel Juárez

**Affiliations:** 1 Centro de Edafología y Biología Aplicada del Segura (CEBAS), CSIC, Departamento de Biología del Estrés y Patología Vegetal, 30100 Murcia, Spain; 2 Centro de Investigación e Innovación Agroalimentaria y Agroambiental (CIAGRO), Universidad Miguel Hernández de Elche, 03312 Orihuela, Spain

**Keywords:** aphids, cucurbits, CABYV, crop management, WMV, viral symptoms

## Abstract

The effectiveness of pest and disease management in crops relies on knowledge about their presence and distribution in crop-producing areas. Aphids and whiteflies are among the main threats to vegetable crops since these hemipterans feed on plants, causing severe damage, and are also able to transmit a large number of devastating plant viral diseases. In particular, the widespread occurrence of aphid-transmitted viruses in cucurbit crops, along with the lack of effective control measures, makes surveillance programs and virus epidemiology necessary for providing sound advice and further integration into the management strategies that can ensure sustainable food production. This review describes the current presence and distribution of aphid-transmitted viruses in cucurbits in Spain, providing valuable epidemiological information, including symptom expressions of virus-infected plants for further surveillance and viral detection. We also provide an overview of the current measures for virus infection prevention and control strategies in cucurbits and indicate the need for further research and innovative strategies against aphid pests and their associated viral diseases.

## 1. An Introduction to Viral Diseases Affecting Cucurbit Crops

Cucurbits are among the most important horticultural vegetables worldwide. The production of these vegetables (melon, watermelon, zucchini, cucumber, and pumpkin, among others) is often seriously affected by several pests and diseases [1,2]. Among them, viral diseases stand out not only for their negative impact on food quality and yield [3,4,5,6,7] but also for the complexity of controlling these diseases in an efficient manner and the rise in major epidemic events when control is deficient [8]. Although cucurbit crops may be grown under protected production systems (tunnels and greenhouses), they are mostly grown in open fields from spring to early autumn in temperate and subtropical regions. Herein, viral diseases that are transmitted by insect vectors are highly relevant, negatively affecting yield and quality of crop production. It has been estimated that plant viruses are responsible for around USD 30 billion in annual losses in crops [9]. Without a reliable estimation of the economic impact of viral diseases on cucurbit crops, there are 28 viral species that have been recently described to be relevant to cucurbit crops in the Mediterranean basin [3,10,11] (Table 1). It is remarkable that most of these viruses are vectored by aphids and whiteflies (order Hemiptera: *Aphidoidea* and *Aleyrodoidea*, respectively) [11,12]. Among them, cucurbit aphid-borne yellows virus (CABYV), cucumber mosaic virus (CMV), watermelon mosaic virus (WMV), and zucchini yellow mosaic virus (ZYMV) have been reported in more than half of the 26 countries of the Mediterranean basin [11,12,13,14,15], whereas the remaining 24 viruses have also been found to cause damages in cucurbit crops, but occasionally either at the local or regional levels. These include whitefly-borne viruses, such as cucurbit yellow stunting disorder virus (CYSDV), and tomato leaf curl New Delhi virus (ToLCNDV), with significant and negative effects on cucurbit production [16,17,18], or viruses that can be transmitted either by leafhoppers, such as the rhabdovirus eggplant mottled dwarf virus (EMDV), or soil fungi, such as the carmovirus melon necrotic spot virus (MNSV). In addition, we also find seed-borne viruses, such as the tobamovirus cucumber green mottle mosaic virus (CGMMV), which is severely affecting cucurbit crops in the Mediterranean basin [3,11]. Among these, cucurbit chlorotic yellows virus (CCYV) is also worth mentioning, as this crinivirus was first described in 2004 in melon plants in Japan, and since then, it has been reported in countries such as Greece, Lebanon, Korea, India, Iran, Sudan, Taiwan, China, and the United States, where it affects different cucurbit crops [10,19,20,21,22,23,24,25,26,27,28], including the recent identification in cucumber, watermelon and zucchini crops in Spain [29,30].

Within the Mediterranean basin, Spain is among the largest producer of cucurbits, with a total of 67,500 ha cultivated in the central and southeastern areas and over 3 million tonnes harvested a year [31]. Our current observations in these crops suggest that the incidence of aphid-transmitted viruses has increased in the last few seasons [32,33,34], concurrently with an increase in the aphid population in the early stages of crop cultivation. Thus, a better understanding of the intricate epidemiology of cucurbit viral diseases will allow the improvement of the management strategies utilized for these pests and the aphid-transmitted viruses in crops [35,36]. Here, we review the occurrence of CABYV, CMV, papaya ringspot virus (PRSV), WMV, Moroccan watermelon mosaic virus (MWMV), and ZYMV in cucurbit crops, providing a brief description of their biology and symptom expression in the major cultivated plant species, and considering their geographical distribution and epidemiological status from 2011 to 2020 in Spain. In addition, current strategies for the prevention and control of these viral diseases are discussed, drawing attention to the need to understand the plant-aphid-virus interplay as a crucial prerequisite for improving and developing innovative and sustainable control strategies for the management of aphid pests and their associated viral diseases in agriculture. This review will contribute to the knowledge of the aphid-borne viruses in cucurbits crops in Spain, as well as to the knowledge of the epidemiological state and management of these viral diseases.

## 2. Description of the Main Aphid-Transmitted Virus in Cucurbits

### 2.1. Cucurbit Aphid-Borne Yellows Virus

**Figure 1:**
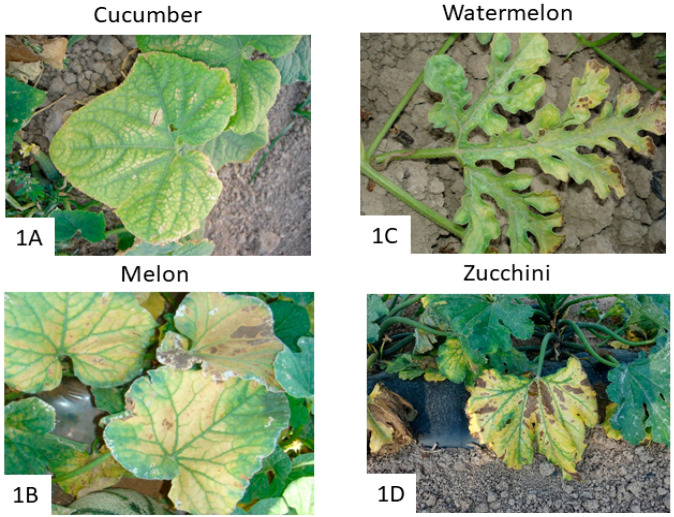
Symptoms of CABYV infection on cucurbit leaves. CABYV-affected plants show yellowing and vein banding symptoms on cucumber leaves (**A**), as well as on melon (**B**), watermelon (**C**), and zucchini (**D**). Additionally, necrotic rings can also be observed on basal leaves of melon (**B**), watermelon (**C**), and zucchini (**D**).

CABYV is a member of the genus *Polerovirus* (family *Luteoviridae*). CABYV particles are icosahedrical with an approximate diameter of 25 nm, and its genome consists of a single-stranded positive-sense RNA molecule of about 5.7 kb [3]. It is limited to phloem tissues in infected plants and is transmitted in a persistent (circulative and non-propagative) manner by the aphid species *Aphis gossypii* Glover, *Myzus persicae* Sulzer, and *Macrosiphum euphorbiae* Thomas [37]. Based on phylogenetic analyses of full-length genome sequences, CABYV has been divided into four groups: Asian, Taiwan, Mediterranean, and recombinants [38,39,40]. CABYV mainly infects cucurbit species such as cucumbers, melons, watermelons, courgettes, pumpkins, and bitter cucumbers, in addition to other cultivated species such as fodder beet *(Beta vulgaris*) and lettuce (*Lactuca sativa*), and a variety of wild plant species: *Papaver rhoeas*, *Ecballium elaterium*, and *Capsella bursa-pastoris*, which could serve as reservoirs for vectors and/or sources of inoculum [11,41,42]. CABYV causes symptoms entirely in cucurbit plant species, causing significant flower abortion. Depending on the plant species, virus isolate, the age of the plant at the time of infection, and environmental conditions, yellowing is the most common symptom induced by CABYV infection, which causes systemic discoloration of the plant (Figure 1). This CABYV yellowing may vary from slight to mild thickening and brittleness, with vein banding leading to green veins (Figure 1A–D). In addition, necrotic rings are often observed on basal leaves due to starch accumulation and dehydration, with subsequent collapse (Figure 1B–D). However, new emerging CABYV variants and mixed infections may ameliorate or exacerbate plant symptoms, frustrating the phytosanitary inspections of cucurbit crops. In fact, a novel CABYV variant has recently been reported in watermelon crops, causing more severe symptoms of yellowing and interveinal chlorotic mottling not only in watermelon plants but also in zucchini and melon plants, as compared to the classical CABYV isolate [34].

### 2.2. Cucumber Mosaic Virus

**Figure 2:**
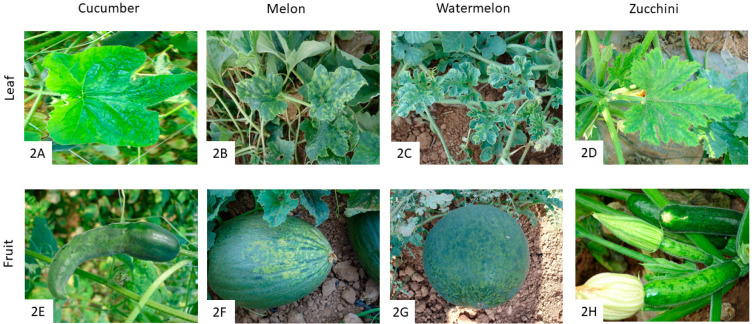
Symptoms of CMV infection on cucurbit leaves and fruits. CMV-affected plants show symptoms of mild mosaics on cucumber leaves (**A**), and yellow or green mosaics, deformation, and enation on cucumber fruits (**E**). In melon, mosaics, chlorotic spots, and curling on leaves (**B**), and mosaic, mottling, and stunting on fruits (**F**) are observed. In watermelon, leaves may also show mottling (**C**), and mosaic, mottling, and bubbling on fruits (**G**). Zucchini leaves show mosaic (**D**), with deformation, mosaic, mottling, and stunting on zucchini fruits (**H**).

CMV belongs to the *Cucumovirus* genus (family *Bromoviridae).* Its particles are roughly spherical and about 30 nm in diameter, and its genome has three-partite, single-stranded RNA molecules, which code for five genes [43]. CMV is transmitted in a stylet-borne non-persistent manner by 70 aphid species, including *M. persicae*, *A. gossypii*, *A. craccivora* Koch, and *A. fabae* Scopeli [44,45,46]. Although, it has been experimentally shown that *A. gossypii*, *A. glycines* Matsumura, *Acyrthosiphon pisum* Harris, and *Therioaphis trifolii* Monell are the most efficient vectors of CMV [47]. In addition, seed transmission of CMV takes place in some species such as spinach, zucchini, bean, and some weedy plants such as wild cucumber (*Echinocystis lobata)* and *Stellaria media* (L.) Vill [43,44,46]. In spite of this, it has been reported that transmission rates are quite low, although higher rates have been reported. For example, transmission rates of up to 21% have been observed in cowpea seeds, which is enough to cause an epidemic [47]. Based on serology, symptomology, host range data, and nucleotide sequence of either CP or the 3′ or 5′ UTR of RNA3, CMV strains have been classified into three subgroups: IA, IB, and II [48]. While Subgroup IA and II have a worldwide distribution, Subgroup IB is mainly present in Asia, although it has also been reported in Spain, Italy, and Greece, with a few isolates in the USA and Brazil [47]. CMV has a broad host range, including monocotyledons and dicotyledons plant species, with more than 1200 known hosts [11,49]. For example, CMV infects plant species belonging to different botanical families, such as *Brassicaceae*, *Solanaceae*, *Papilionaceae*, and *Cucurbitaceae*, including weeds and ornamental plants such as *Chenopodium amaranticolor*, *Datura innoxia*, *Hibiscus rosa* or *Salvia splendens* [49,50,51]. Recently, it has also been reported to infect *Ocimum gratissimum* L. in Nigeria [52] and *Tylophora indica* in India [53]. In Spain, CMV has been found to be widely distributed in cucumber, melon, pumpkin, zucchini, watermelon, tomato, pepper, aubergine, tobacco, beans, celery, and borage horticultural crops, among others [42,47,50,54]. The most common symptom of CMV infection is the presence of mosaics on the leaves (Figure 2), although symptom expression varies according to the plant species, virus isolate, the age of the plant at the time of infection, and environmental conditions [55]. Moreover, CMV symptoms can be modified by the presence of RNA satellites [56]. For example, a satellite RNA, CARNA-5, has been associated with CMV, and in the case of tomato infection, it becomes lethal to the plant [47]. In our observations of CMV infection in cucurbits, mosaic patterns were commonly observed on leaves and fruits (Figure 2A–H). For instance, mild mosaics may appear in cucumber, melon, watermelon, and zucchini leaves (Figure 2A–D, respectively), including chlorotic spots and curling on melon leaves (Figure 2B), as well as yellow mottling on watermelon leaves (Figure 2C). CMV symptoms on fruits generally consist of deformation, mosaic, mottling, and stunting (Figure 2E–H), with yellow or green mosaics and enation (Figure 2E,H), and even bubbling (Figure 2G,H).

### 2.3. Papaya Ringspot Virus

**Figure 3:**
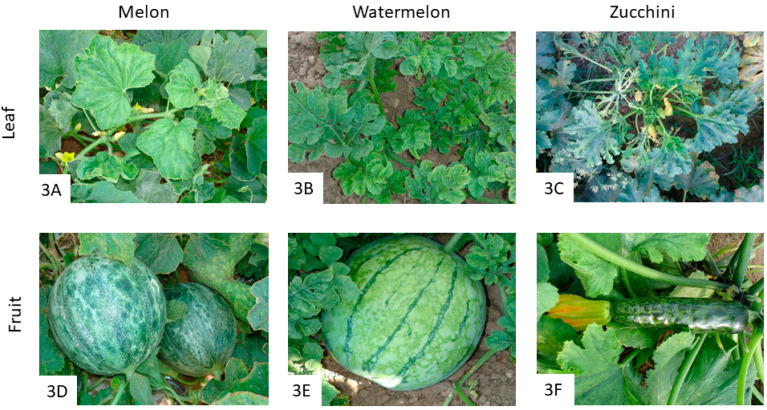
Symptoms of PRSV infection on cucurbit leaves and fruits. PRSV-affected plants show symptoms of chlorosis and mosaics, including curling and malformations on melon (**A**), watermelon (**B**), and zucchini (**C**) leaves. Affected fruits show deformation and discoloration, along with dark green blistering, distortion banding, and ringspots in melon (**D**), watermelon (**E**), and zucchini (**F**).

PRSV belongs to the genus *Potyvirus* (family *Potyviridae*), and it is composed of flexuous particles of about 780 nm. Its genome consists of a unipartite linear single-stranded positive-sense RNA of 10 kb [3,57]. PRSV is transmitted by more than 20 aphid species in a non-persistent manner, including *M. persicae*, *Aulacorthum solani* Kaltenbach, *A. craccivora*, *A. gossypii*, *M. euphorbiae*, *Toxoptera aurantii* Boyer de Fonscolombe, and *Rhopalosiphum maidis* Fitch [58]. There is experimental evidence that suggests that PRSV could be transmitted mechanically during the harvesting process or mechanically by contact between plant leaves [57]. In fact, there is one study in which the authors performed mechanical inoculations of PRSV in different cucurbit plants, and they observed that zucchini squash was the most susceptible species, followed by watermelon and cucumber, while the pumpkin was not infected by mechanical inoculations of PRSV [59]. Serologically, PRSV has been divided into two biotypes; the papaya-infecting biotype P (PRSV-P) and the cucurbit-infecting biotype W (PRSV-W). PRSV-P was first described from papaya in Hawaii, and PRSV-W was formerly known as watermelon mosaic virus 1 (WMV-1), which might have appeared in Australia 20 years before PRSV-P, suggesting that PRSV-P may have originated from PRSV-W [60]. PRSV has a very restricted host range, mainly infecting members of the *Cucurbitaceae* family, with the exception of the papaya from which it takes its name, and some non-cucurbit species such as *Chenopodium* [11]. In this sense, while PRSV-W isolates infect plants from *Cucurbitaceae* and *Chenopodiaceae* families, PRSV-P isolates also infect papaya plants [57]. Symptoms associated with PRSV may vary depending on the plant species. In papaya, PRSV-infected plants show typical symptoms of vein-clearing, yellow mottling, rough surface, and bristly leaf margins, stunting, and partial or complete cessation of fruiting [61]. In cucurbits, PRSV symptoms are similar to those found on papaya, consisting of chlorosis and mosaics on leaves, with curling and malformations (Figure 3A–C). In fruits, deformation, discoloration, and flower abortion are the major PRSV symptoms in melon, watermelon, and zucchini (Figure 3D–F), with dark green blisters, distortion banding, and ringspots as well. In the case of some members of the family *Chenopodiaceae*, symptoms are milder than in papaya or cucurbits crops [57].

### 2.4. Watermelon Mosaic Virus

**Figure 4:**
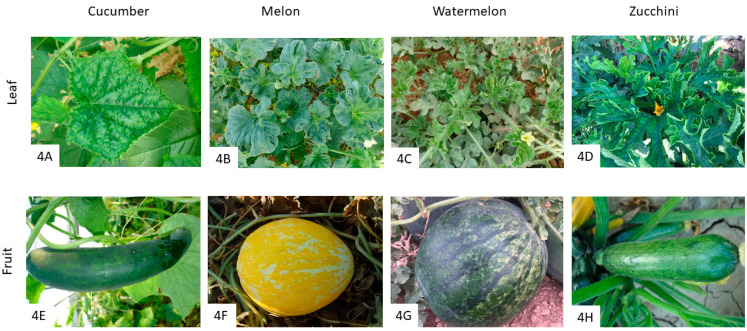
Symptoms of WMV infection on cucurbit leaves and fruits. Plants affected by WMV show mosaic symptoms, including sawn edges, vein clearing, and banding in cucumber leaves (**A**). In melon, mosaic, mottling, and leaf deformation are observed on leaves (**B**), as well as on watermelon (**C**) and zucchini (**D**) leaves. Affected fruits show yellow and green mosaics, with enation and deformations in cucumber (**E**), colorlessness on melon (**F**), and blistering on watermelon (**G**) and zucchini (**H**) fruits. Similar symptoms are observed in plants affected by MWMV.

WMV is another potyvirus with flexuous and filiform particles of about 760 nm in length [3]. Its genome consists of a single-stranded positive-sense RNA genome, comprising a unique open reading frame of about 10 kb that encodes for a polyprotein [62]. WMV can be transmitted in a non-persistent manner by at least 35 species of aphids in 19 genera, with the most important being *A. citricola* V. der G., *A. craccivora*, *A. gossypii*, *A. solani*, *M. euphorbiae*, *M. persicae*, and *T. citricidus* Kirkaldy. WMV has not been reported to be seed transmitted in cucumber, watermelon, melon, or zucchini [11], although it can be transmitted mechanically in laboratory conditions and through vegetative propagation in vanilla [63,64], and other species such as *C. quinoa*, *C. amaranticolor*, *Nicotiana benthamiana*, *N. glutinosa*, and *N. tabacum*, as well as cucurbits species and some legumes such as *Trifolium incarnatum*, *T. subterraneum*, *Pisum sativum* and *Vicia faba* [65,66]. As stated before, WMV-1 is considered the W strain of PRSV, and WMV-2 is referred to as WMV [64]. WMV isolates have been classified into three molecular groups based on the amino acid sequences of the coat protein N-terminal region: Group 1 isolates, which are also known as classical (CL) isolates, Group 2 isolates, and Group 3 emerging isolates (EM), with its isolates divided into four genetic subgroups (EM1, 2, 3, and 4). These subgroups were associated with a different geographic distribution in southern France, where it was observed that wherever EM1 and EM2 were present, EM3 and EM4 were absent [11,67,68]. It has been shown that the accumulation and transmission efficiency of EM isolates is higher than CL and Group two isolates, which could explain the displacement of both groups by the EM group in the Mediterranean basin [63,69]. WMV has a wide host range, infecting over 170 species of monocotyledonous and dicotyledonous plant species, including cultivated cucurbits and peas, beans, spinach, and vanilla crops, as well as weeds (senecio, shepherd’s purse, nettle or fumaria, among others) [63,64]. WMV causes typical potyvirus symptoms, although it depends on the plant species. In cucurbit leaves, mosaic patterns are commonly observed (Figure 4A–D), with vein clearing and banding in cucumber (Figure 4A), including mottling mosaics and leaf deformation in melon, watermelon, and zucchini (Figure 4B–D). In fruits, yellow and green mosaics are observed (Figure 4E–H), with enation (Figure 4E), colorless (Figure 4F,G), and blisters (Figure 4H).

### 2.5. Moroccan Watermelon Mosaic Virus

MWMV is a potyvirus that results from PRSV at the genetic level, with a single-stranded RNA genome of about 10 kb [70]. Similar to WMV, this virus can be transmitted mechanically, but it is naturally transmitted by aphids in a non-persistent manner [71]. *M. persicae*, *A. gossypii*, and *A. spiraecola* Patch can efficiently transmit MWMV, with *M. persicae* and the subspecies *M. persicae nicotianae* being the most competent vectors in cucurbit crops [71]. Phylogenetic analyses have shown three genetic groups (A-C): Clade A comprises isolates from Tunisia and Europe; clade B, isolates from South Africa; and clade C, isolates from Central Africa [72]. Cucurbits are the main hosts of MWMV [64], in addition to other crops, such as papaya (*Carica papaya*) [73], and wild species, such as *Chenopodium amaranticolor*, *C. quinoa*, and *Ranunculus sardous* [72]. MWMV symptoms are similar to those of WMV, resulting a severe vein clearing and banding with dark green mosaics, including sawn edges on leaves (Figure 4A–D). Fruit symptoms consist of deformations (Figure 4E), discoloration (Figure 4F), mosaics, and blistering (Figure 4G,H, respectively).

### 2.6. Zucchini Yellow Mosaic Virus 

**Figure 5:**
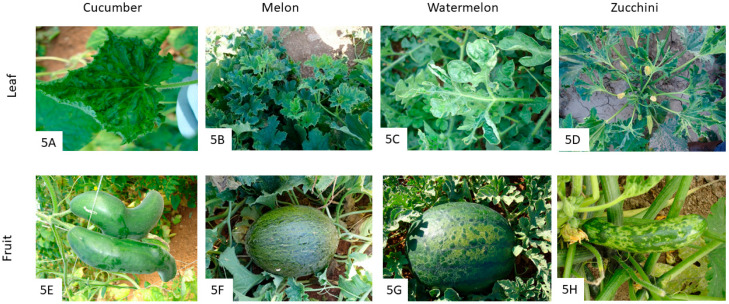
Symptoms of ZYMV infection on cucurbit leaves and fruits. ZYMV-affected plants show symptoms of vein clearing, deformation, mosaics with bubbling, blistering, sawn edges, and enation observed in cucumber (**A**), melon (**B**), watermelon (**C**), and zucchini (**D**) leaves. Affected fruits show enation, mosaic, or necrotic cracks, with severe deformations on cucumber (**E**) and melon (**F**), including bubbling in watermelon (**G**) and zucchini (**H**) fruits.

ZYMV also belongs to the genus *Potyvirus*, with flexuous particles of about 750 nm in length. Its genome consists of a single-stranded RNA of about 9.6 kb [74]. ZYMV is transmitted in a non-persistent manner by at least 26 aphid species. However, only a few aphid species have been tested for their transmission efficiency, apart from *A. gossypii*, *M. persicae*, *A. craccivora*, and *M. euphorbiae* [11,64]. Three genetic groups have been described in the ZYMV population: Group A, which is in turn divided into six clusters, and Group B and C. While isolates from group A have only been detected in the Mediterranean basin, isolates from groups B and C have been detected in Vietnam and China, as well as isolates from group C in Poland [11]. ZYMV has a restricted host range, primarily infecting cultivated or wild cucurbit plant species, in addition to a few weed species of the *Delphinium*, *Begonia*, and *Altheae* genera [64]. Symptom expressions of ZYMV in *Chenopodium amaranticolor* and *C. quinoa* range from local lesions or latent infections [74]. In cucurbits, ZYMV symptoms may include vein clearing, deformation, and mosaics, with bubbling, blistering, sawn edges, and enation (Figure 5A–D). In fruits, enation, mosaic, or necrotic cracks appear to be common (Figure 5F–H), with severe deformations (Figure 5E) and bubbling (Figure 5G,H).

## 3. Geographical Distribution and Occurrence of Aphid-Transmitted Viruses

Since aphid-borne viruses are widely distributed in cucurbit-producing areas, it is crucial to determine which virus species are present and which factors lead to their prevalence in order to address successful crop management. On the one hand, the lack of reliable viral detection during phytosanitary inspections is a risk factor. Monitoring and surveillance programs must be accompanied by appropriate serological or molecular diagnostic methods [75], in addition to the characterization of the genetic diversity and population structure since novel variants can emerge. The monitoring based on symptom expression is often technically challenging, whereas different viruses induce similar symptoms, some plant species may be asymptomatic, or symptoms can even be mistaken for physiological disorders or nutrient deficiencies [76]. Serological methods, such as enzyme-linked immunosorbent assay (ELISA) and its variants, have been used for decades, allowing the identification and detection of viruses in a fast and easy manner [77]. Molecular methods, such as polymerase chain reaction (PCR) and all its variants, DNA microarray, as well as tissue-print and dot-blot hybridization, have high sensitivities and reasonable costs [75,77,78]. Nevertheless, current and innovative techniques, such as metagenomics high-throughput sequencing (HTS), CRISPR/Cas12, or machine learning (ML) approaches, have created a revolution in the detection of multiple viruses that are either known or unknown [79,80,81].

On the other hand, the combination of multiple abiotic and biotic factors can be responsible for the prevalence of viral diseases [82,83,84,85,86,87]. In cucurbit crops, the commercial exchanges of seeds, plants, or fruits are associated with the geographical distribution of these viral diseases. However, the aphid vector abundance and transmission efficiency, along with the presence of alternative host reservoirs, including other cultivated crops and wild plant host species that may overlap at spatio-temporal scales, can underlie the plant viral distribution [88,89]. Additionally, agricultural ecosystems composed of highly dense and genetically uniform host populations can favor the emergence and prevalence of viral diseases [88], where socio-cultural changes and alterations of the growing agricultural production can create fragmentations in the distribution of vectors, generating changes over the viral populations. Furthermore, several studies have reported how host preference and vector behavior influence the transmission and spread of plant viruses [35,90,91], and in turn, how environmental conditions can affect the virus prevalence and vector transmission, as climate change may have a significant influence on virus pathogenicity, ecology, and evolution [92,93]. Attention should be paid to the fact that the aforementioned factors can also be influenced by the co-occurrence of related and/or unrelated viruses in the same plant, which may consequently alter virus accumulation and transmission rates [94,95,96]. In this case, mixed infections of aphid-borne viruses in the same plant can be found, and some examples are gathered in Supplementary Table S1. Therefore, the lack of early and appropriate virus detection, along with the variation between several abiotic and biotic factors, have an influence on the occurrence of viral diseases, underlie their prevalence, and shape the evolutionary dynamics of each viral population.

### 3.1. Cucurbit Aphid-Borne Yellows Virus

**Figure 6:**
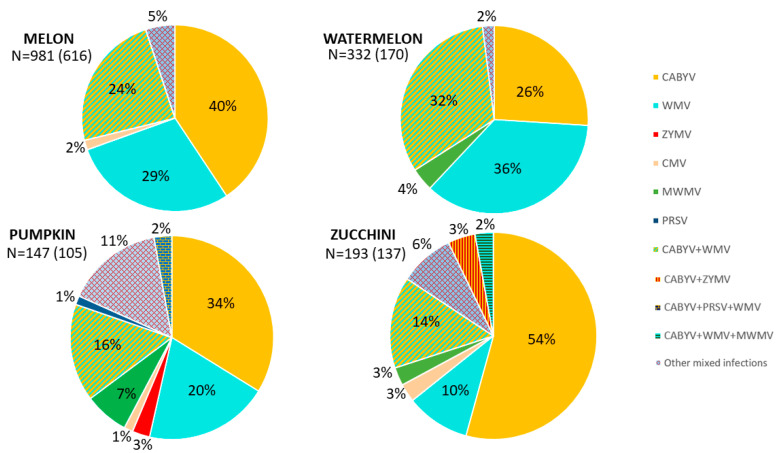
Occurrence and distribution of cucurbit aphid-transmitted virus in Spain. The proportion of aphid-transmitted cucurbit viruses from symptomatic cucurbit cultivated plants over 12 cucurbit growing seasons (2011 to 2022). These epidemiological data came from a long-term monitoring study (2011 to 2020) of aphid-borne viral diseases [32,33,34], including the unpublished data from the last two growing seasons (2021 and 2022). The proportion of each virus is represented in each slice, with a color assigned for each virus and indicated in the legend. Single infection is represented by a solid color and mixed infection by a stripped color, combining the color of each virus in a single infection. N = total samples (total positive samples detected). CABYV and WMV are the most prevalent aphid-transmitted viruses affecting cucurbit crops in Spain.

CABYV is widespread in Europe, especially in the Mediterranean region, although it also occurs in Asia, parts of Africa, and North America. CABYV was first described in cucurbit production areas of France in 1992 [97]. Since then, it has spread to Cyprus, the Czech Republic, Greece, Italy (including Sicily), Spain, and Tunisia [3,5,98,99,100], with current detections in Poland, Slovenia, Germany, and Bulgaria [101,102,103,104]. In Spain, CABYV was first identified in 2004 in the Murcia region in melon and zucchini plants [14], and since then, it has become one of the most prevalent viruses affecting cucurbit crops reaching incidences of 83% and 66% for melon and squash samples, respectively [14,32,34]. Indeed, after combining the epidemiological data from our long-term monitoring studies (2011 to 2020) for aphid-borne viral diseases [32,33,34], including the last two growing seasons (2021 and 2022), CABYV was found to be the most prevalent virus in cucurbit crops. It was detected in a high proportion of symptomatic samples of zucchini (54%), melon (40%), pumpkin (34%), and watermelon (26%) in single infections (Figure 6). Furthermore, there was also a high proportion of samples with CABYV and WMV in mixed infections in melon (24%), watermelon (32%), pumpkin (16%), and zucchini (14%) (Figure 6). The combination of CABYV with other viruses, such as ZYMV, PRSV, and MWMV, was present in low occurrence (<3%) and only detected in pumpkin and zucchini (Figure 6). In addition, CABYV is often found in the presence of other viruses in the same plant (Supplementary Table S1).

### 3.2. Cucumber Mosaic Virus

CMV was first described in 1916, resulting in one of the viruses that most frequently affects cucurbit crops worldwide [43]. Since CMV also affects major annual crops, it is widely distributed in Argentina, eastern China, Croatia, France, Egypt, Greece, Israel, Italy, Japan, Poland, Portugal, Sweden, and the northeastern US [105,106,107,108]. According to molecular analyses of the genomic RNAs, CMV has been classified into two extremely heterogeneous subgroups [108]. Subgroup I has been reported in both temperature and tropical regions and is divided into two subgroups (IA and IB) based on analysis of RNA 3 open reading frames, whereas Subgroup II has been reported in cooler areas and seasons of temperate regions being Subgroup I more heterogeneous than Subgroup II [108]. In the Mediterranean basin, CMV is present in all countries [11]. In Spain, it was reported for the first time in 1995 in melon crops [109], with a high incidence in some cases [110,111]. Since then, the genetic structure of CMV in different locations in Spain has been studied, showing that most CMV isolates belong to Subgroup I, which has a wide distribution in cucurbit crops and others, such as tomato crops in simple and mixed infections [112]. Moreover, studies in other hosts, such as weeds, have shown that from 1999 to 2002, CMV was detected with a maximum incidence of 30% in weed hosts and melon in Central Spain [50]. Systematic surveys in 2003 and 2004 in open field melon and squash in the Murcia Province showed that the incidence of CMV was less than 6% and 1.5% in melon and squash samples, respectively [105]. In the Valencian Community, from 2005 to 2006, it was shown that the CMV incidence was less than 10% of the total plants from different cucurbits (melon, watermelon, pumpkin, cucumber, and squash) [69]. In a cucurbit survey study in 2018, from open fields in the Murcia Province, Castilla-La Mancha, and the Valencian Community, it was reported that CMV had a low incidence and that the CMV sequences showed a high identity between themselves and with European isolates [15]. This is in accordance with our long-term monitoring program from 2011 to 2022, which showed that CMV was only detected in symptomatic samples of melon (2%), pumpkin (1%), and zucchini (3%) [32,33,34] (Figure 6).

### 3.3. Papaya Ringspot Virus

PRSV is the cause of one of the most serious diseases affecting cucurbit crops in the Southern United States and the Antilles, although it also occurs in several Mediterranean countries (Bulgaria, Cyprus, France, Israel, Italy, Lebanon, Spain, Syria, Tunisia and Turkey) [11,57], and has even been detected in pumpkin in Sudan, zucchini in Poland and Morocco, and papaya in Argentina for the first time [113,114,115,116]. In Spain, PRSV was detected for the first time in zucchini in Malaga in 1985 [117]. In 1995, PRSV was present in melon crops in Spain with a lower incidence [109], and since then, PRSV has been present in different cucurbit crops with variable incidences between regions, in both simple and mixed infection with other viruses year by year [14,33,34,69]. Although with a low frequency in Spanish crops, PRSV has been detected in pumpkin samples (1%), with the last detection in 2019 under mixed infections with CABYV and WMV [32,33,34] (Figure 6).

### 3.4. Watermelon Mosaic Virus

WMV is widely distributed in cucurbit crops worldwide [63]. It was reported in most countries in the Mediterranean region, the first time being in 1963 in Israel. Since then, it has also been found in Italy, Tunisia, and France, among others [11], with the most recent detections in Bosnia, Herzegovina, and Poland [118,119]. In Spain, WMV is among the most widely distributed cucurbit viruses [110,117,120] and has become highly prevalent in melon and zucchini crops [14,69,109,111]. In melon crops, WMV was the most frequently found virus in surveys carried out in 1995 and 1996, although it is known that WMV has been present in field-grown melons in Spain since 1991 [109]. Cucurbit crop surveys carried out in eastern Spain in 2005 and 2006 showed that WMV was present in 27% of samples and was also detected in 36% of samples under mixed infections [69]. However, during the 2003 and 2005 seasons in Murcia, WMV incidence was 21% and 6% in melon and squash crops, respectively, where mixed infections of CABYV + WMV were present with a high frequency (18%) in melon crops [14]. Thus, and similar to the CABYV assessment from 2011 to 2022, WMV is well-recognized as a prevalent virus in melon, watermelon, pumpkin, and zucchini crops, with a higher occurrence in watermelon (36%), as compared to melon (29%), pumpkin (20%), and zucchini (10%) plants, where it is lower in single infections [32,33,34] (Figure 6). There was also a high frequency of mixed infections between WMV and CABYV, as mentioned above (Figure 6). The combination of WMV with others, such as CABYV and PRSV, or CABYV and MWMV in a triple infection, was found in a low occurrence (<2%) in pumpkin and zucchini (Figure 6).

### 3.5. Moroccan Watermelon Mosaic Virus

The initial identification and characterization of a novel WMV isolate from Morocco in 1974 resulted in the consideration of this MWMV as an emergent threat to cucurbit crops in the Mediterranean basin [11,121]. It has been described as causing damage to cucurbits crops in all producing regions, such as South, Central, and North Africa, and also European countries such as Italy, Greece, France, Portugal, and Spain [72,121,122,123,124]. Additionally, it has been reported in the Democratic Republic of Congo in papaya, in Turkey in squash, and in Iran and Greece, in zucchini [73,124,125,126]. In Spain, it was described for the first time in zucchini crops [121], and since then, its occurrence has been low, although there was a resurgence in 2018 in watermelon and pumpkin crops in the cucurbit-producing areas of Murcia, Alicante, and Castilla-La Mancha [33]. MWMV was present in 4%, 7%, and 3% of symptomatic samples of watermelon, pumpkin, and zucchini, respectively, with no detection in melon [32,33,34] (Figure 6).

### 3.6. Zucchini Yellow Mosaic Virus

ZYMV was reported for the first time in 1973 in zucchini plants in Italy, and since then, it has been reported in 18 Mediterranean countries, including France, Israel, Egypt, and Turkey [11,64], as well as in watermelon crops in Serbia, and Bosnia and Herzegovina [127,128]. It has also been reported in other cucurbit species, such as bitter melon in South Korea [129] and gherkin in India [130]. In Spain, it is known that ZYMV has been present in melon crops since 1991 [109]. Since then, surveys carried out in different cucurbit crops over the years showed that ZYMV had a low incidence of less than 10% [14,69,117], as well as in mixed infection with other viruses [15]. From our long-term epidemiological data, ZYMV was found in a low occurrence in pumpkin crops (3%) from 2011 to 2022 and also in mixed infections combined with other aphid-transmitted viruses in melon, watermelon, and zucchini in a low occurrence (<1%) [32,33,34] (Figure 6). It is worth mentioning that the combination of ZYMV and CABYV was present in 3% of zucchini samples (Figure 6).

## 4. Overview of the Management of Aphid-Transmitted Viral Diseases

Despite advances in plant virus control and efforts to reduce or delay the spread and dispersal of the aphid-transmitted viruses described above, they are widely distributed, causing severe losses in yield and quality on cucurbit crops. Current methods for controlling these aphid-viral diseases result from preventive measures [131]. These include the use of plant material that is resistant and/or tolerant to these viruses, in addition to the use of virus-free material combined with cultural practices, such as weed control and crop rotation, as well as controlling vector-mediated transmission. These control options have been comprehensively described by other reviews [3,13,36,132,133,134,135,136,137], and even novel techniques based on nanotechnology have been recently reported for the management of plant virus diseases [138,139,140]. In cucurbit crops, it is important to consider that these crops grow in open fields and temperate growing areas, where the aphid-vector populations are also major pests that are difficult to control. This underscores a complex scenario where a profound understanding of the interplay between plant-virus-vector is crucial for the prevention and control of aphid pests and their associated viral diseases. In the following sections, we provide an overview of the general and local control measures for these aphid-transmitted viruses.

First, the most effective management option for virus control on plants is the use of resistant cultivars [141,142]. In this sense, genetic resistance and tolerance have been reported in a few melon, watermelon, squash, and cucumber lines through organogenesis or embryogenesis using agrobacterium-mediated transformation, mitigating symptom expression against ZYMV, WMV, CMV, and PRSV-W [141,142,143]. Potential sources of resistance to PRSV-W, WMV, and ZYMV have also been reported from melon accessions (C-189, C-105, C-885, C-769) and from wild relatives [144]. There are commercially-grown hybrid cultivars of melon with tolerance to aphids, in addition to cultivars carrying the *vat* gene, which confers resistance to both *A. gossypii* and the viruses it transmits [145]. With the means to edit cucurbit genomes directly through the revolutionary CRISPR/Cas9 approach [146], cucumber lines with mutations that disrupt the eIF4E interaction with viral proteins, which is associated with recessive resistance [147], have been reported and tested against CVYV, ZYMV, PRSV-W, CMV and CGMMV [148], and melon lines against MWMV [149]. This technology is reducing the timeframe needed to obtain cultivars carrying traits of interest and is further underway to incorporate viral disease control into plant crops. Additionally, despite the fact that these viruses are mainly aphid-transmitted, in some cases seed transmission may occur, and this is why seed certification programs are mandatory to control the primary sources of viral inoculum and contribute greatly to success in controlling viral diseases [150]. Additionally, cultural practices by the use of physical barriers can readily diminish the presence of insects in the growing area. For example, in open-field production, the use of floating row covers and colored plastic mulches affects the abundance and presence of aphid species in the field production of watermelon [151]. This covering could prevent the crop, with moderate effectivity, especially against viruses that are transmitted in a non-persistent manner. In greenhouse production, the use of sticky insect traps and dense netting must be considered [134,152]. Another cultural practice is the use of border plant cultivation, known as trap crops, which can limit the spread of insect-vector-protecting cucurbit crops [13,153]. For example, in the USA, intercropping of pumpkin and sorghum can reduce the incidence of PRSV and WMV from 96% to 43%, while intercropping with other crops, such as soybean and peanut, was less effective than sorghum [154]. Additionally, tilling and the elimination of symptomatic plants or crop residues from previous seasons, including the alternative wild plants or others that can act as reservoirs and/or sources of viral inoculum, must be considered [155]. For example, it has been shown that viral incidence rates in wild plants could be one of the most important factors in explaining the epidemiological patterns of ZYMV and WMV [13,47]. Furthermore, modifications in the growing crop system may also lead to alterations in the viral population dynamics, increasing the risks of the virus emergence or outbreaks in new geographical regions [8,87]. For example, the intensification of vegetable production, increased connectivity between plant populations, along with global warming are considered to increase the risk of virus outbreaks [8,93]. In this sense, the speeding up replacement from traditional to organic/ecological crop production is thought to temporally affect the aphid pests and viral disease situation and requires a better understanding of the aphid-virus-plant interaction to understand the intricated epidemiology of the cucurbit viral diseases and to develop innovative strategies that replace the use of chemical pesticides for managing aphid-transmitted viruses in crops.

Beyond the preventive measures and cultural practices, it is worth considering that particular treatments with mineral oil sprays in the early stages of cultivation have been shown to interfere with the transmission of certain non-persistent viruses [11]. For example, there is evidence that the use of silver nanoparticles or nanomaterials by direct application to soil, seeds, or plant foliage, can have an impact on the bean yellow mosaic virus (BYMV) due to their antiviral activity [140]. In addition, in the early stages of plant cultivation, the implementation of a mild natural virus into the plant by mechanical inoculation to protect commercial crops from the same virulent virus species, known as cross-protection, has also been applied in cucurbits to minimize the risks from the ZYMV [156] and PRSV [157] diseases. However, the feasibility of the use of nanoparticles and cross-protection against insect-vectored viruses remains unknown. Finally, aphid population control is the major strategy utilized to manage these pests and to avoid the spread of viral diseases in cucurbit crops. It is thought that the efficiency of pesticide-based control may vary depending on the aphid-vector acquisition and transmission. While it could be relatively effective against persistently transmitted viruses, such as CABYV, which needs long-time acquisition and retention periods in the aphid-vector, it may have a limited effect against the non-persistently transmitted viruses (CMV, WMV, MWMV, ZYMV, and PRSV). Additionally, phytosanitary measures must be considered not only to prevent the primary plant inoculations but also to constrain the development of aphid colonies in the crop, which may help with the secondary infections and viral spread to the rest of the plot. Nevertheless, it should be noted that aphid species may become resistant to chemical compounds [153]. In fact, in accordance with the EU pesticide legislation 1107/2009 on Plant Protection Products, the negative effects caused by chemical pesticide applications, the increasing production of organic/ecological vegetables and the lack of effective methods to protect crops from aphids, makes it necessary to integrate all available measures and to continue research to find innovative and sustainable strategies that protect crops from pests and diseases.

## Figures and Tables

**Table 1:** Relevant viral species known to affect cucurbit crops in the Mediterranean basin [3,11]. There are 28 virus species, which have been classified according to the vector transmission and taxonomic genus. The significant number of aphid-transmitted viruses affecting cucurbit crops is remarkable. The viruses described in this review are indicated in bold.

TRANSMISSION	GENUS	VIRUS
APHID-BORNE VIRUSES	POTYVIRUS	Algerian watermelon mosaic virus (AWMV)
		Clover yellow vein virus (ClYVV)
		Melon vein-banding mosaic virus (MVBMV)
		Telfairia mosaic virus (TeMV)
		**Papaya ringspot virus (PRSV)**
		Turnip mosaic virus (TuMV)
		**Moroccan watermelon mosaic virus (MWMV)**
		Watermelon leaf mottle virus (WLMV)
		**Watermelon mosaic virus (WMV)**
		Zucchini yellow fleck virus (ZYFV)
		**Zucchini yellow mosaic virus (ZYMV)**
	CUCUMOVIRUS	**Cucumber mosaic virus (CMV)**
	POLEROVIRUS	**Cucurbit aphid-borne yellows virus (CABYV)**
	MASTREVIRUS	Chickpea chlorotic dwarf virus (CpCDV)
WHITEFLY-BORNE VIRUSES	CRINIVIRUS	Beet pseudoyellows virus (BPYV)
		Cucurbit yellow stunting disorder virus (CYSDV)
	IPOMOVIRUS	Cucumber vein yellowing virus (CVYV)
	BEGOMOVIRUS	Squash leaf curl virus (SLCV)
		Tomato leaf curl New Delhi virus (ToLCNDV)
		Watermelon chlorotic stunt virus (WmCSV)
FUNGUS-BORNE VIRUSES	CARMOVIRUS	Cucumber soil-borne virus (CuSBV)
		Melon necrotic spot virus (MNSV)
	AUREUSVIRUS	Cucumber leaf spot virus (CLSV)
	NECROVIRUS	Tobacco necrosis virus (TNV)
SEED-BORNE VIRUSES	TOBAMOVIRUS	Cucumber fruit mottle mosaic virus (CFMMV)
		Cucumber green mottle mosaic virus (CGMMV)
BEETLE-BORNE VIRUSES	COMOVIRUS	Squash mosaic virus (SqMV)
LEAFHOPPER-BORNE VIRUSES	RHABDOVIRUS	Eggplant mottled dwarf virus (EMDV)

## Data Availability

Not applicable.

## Supplementary Materials

The following supporting information can be downloaded at: https://www.mdpi.com/article/10.3390/pathogens12030422/s1, Table S1: Viral species combination that has been reported in mixed infections, including those particular aphid-borne viruses that have been described in this review. Refs. [158,159,160,161,162,163,164,165,166,167,168,169,170,171,172,173,174,175,176,177,178,179,180,181,182,183,184,185] are cited in Supplementary Materials.
