# Supplementary material for: Occurrence, Distribution, and Management of Aphid-Transmitted Viruses in Cucurbits in Spain
Source: Pathogens. 2023 Mar 7;12(3):422. doi: 10.3390/pathogens12030422 (PMC10057868; doi:10.3390/pathogens12030422)
Supplement: Supplementary file 1 [file pathogens-12-00422-s001.zip › pathogens-2218294-supplementary.pdf]

Table S1. Besides the viral species that can lead to mixed infections in plants, which have been reviewed in Moreno and López-Moya, 2020 [1], the following list covers further mixed infections between those particular aphid-borne viruses that have been described in this review and other viral species found in the literature.

APHID-BORNE VIRUSES	MIXED INFECTIONS	CITATION
CABYV	Beet pseudoyellows virus (BPYV)	[2]
	Cowpea aphid-borne mosaic virus (CABMV)	[3]
	Cucumber green mottle mosaic virus (CGMMV)	[4]
	Cucumber leaf spot virus (CLSV)	[5]
	Cucumber mosaic virus (CMV)	[6]
	Cucurbit chlorotic yellow virus (CCYV)	[6]
	Cucumber vein yellowing virus (CVYV)	[2]
	Cucurbit yellow stunting disorder virus (CYSDV)	[2]
	Melon aphid-borne yellows virus (MABYV)	[7]
	Melon necrotic spot virus (MNSV)	[2]
	Moroccan watermelon mosaic virus (MWMV)	[8]
	Papaya ringsplot virus (PRSV)	[2]
	Suakwa aphid-borne yellows virus (SABYV)	[9]
	Tobacco mosaic virus (TMV)	[10]
	Watermelon mosaic virus (WMV)	[2]
	Zucchini yellow mosaic virus (ZYMV)	[6]
CMV	Alfalfa mosaic virus (AMV)	[11]
	Broad bean wilt virus 2 (BBWV2)	[12]
	Bean common mosaic virus (BCMV)	[6]
	Bean yellow mosaic virus (BYMV)	[13]
	Cucurbit aphid-borne yellows virus (CABYV)	[6]
	Canna yellow mottle virus (CaYMV)	[13]
	Cucumber green mottle mosaic virus (CGMMV)	[4]
	Cowpea mottle virus (CMeV)	[14]
	Cowpea mild mottle virus (CPMMV)	[15]
	Cowpea mottle virus (CPMoV)	[15]
	Cucurbit chlorotic yellow virus (CCYV)	[6]
	Cucumber vein yellowing virus (CVYV)	[2]
	Melon necrotic spot virus (MNSV)	[2]
	Pepino mosaic virus (PepMV)	[16]
	Pepper mottle virus (PepMoV)	[17]
	Papaya ringsplot virus (PRSV)	[18]
	Pepper veinal mottle virus (PVMV)	[19]
	Potato virus Y (PVY)	[11]
	Southern bean mosaic virus (SBMV)	[15]
	Southern tomato virus (STV)	[16]
	Tobacco mosaic virus (TMV)	[11]
	Tomato mosaic virus (ToMV)	[11]
	Tomato torrado virus (ToTV)	[20]
	Tomato spotted wilt virus (TSWV)	[11]

	Tomato yellow ring virus (TYRV)	[5]
	Turnip mosaic virus (TuMV)	[21]
	Watermelon mosaic virus (WMV)	[18]
	Zucchini yellow mosaic virus (ZYMV)	[6]
PRSV	Cucurbit aphid-borne yellows virus (CABYV)	[2]
	Cucumber mosaic virus (CMV)	[2]
	Cucumber vein yellowing virus (CVYV)	[2]
	Lettuce chlorosis virus (LCV)	[22]
	Melon necrotic spot virus (MNSV)	[2]
	Papaya mosaic virus (PapMV)	[23]
	Papaya leaf distortion mosaic virus (PLDMV)	[24]
	Squash mosaic virus (SqMV)	[25]
	Tobacco mosaic virus (TMV)	[10]
	Tomato yellow leaf curl virus-IL (TYLCV)	[22]
	Watermelon mosaic virus (WMV)	[26]
	Zucchini yellow mosaic virus (ZYMV)	[26]
WMV	Beet yellows virus (BYV)	[4]
	Cucurbit aphid-borne yellows virus (CABYV)	[2]
	Cucumber green mottle mosaic virus (CGMMV)	[4]
	Cucumber mosaic virus (CMV)	[18]
	Cucumber vein yellowing virus (CVYV)	[2]
	Cucurbit yellow stunting disorder virus (CYSDV)	[27]
	Melon necrotic spot virus (MNSV)	[2]
	Moroccan watermelon mosaic virus (MWMV)	[28]
	Papaya ringsplot virus (PRSV)	[26]
	Squash mosaic virus (SqMV)	[25]
	Tomato ringspot virus (ToRSV)	[29]
	Zucchini yellow mosaic virus (ZYMV)	[26]
MWMV	Cucurbit aphid-borne yellows virus (CABYV)	[8]
	Cowpea mild motter virus (CpMMV)	[30]
	Cucumber vein-clearing virus (CuVCV)	[30]
	Papaya virus A (PaVA)	[31]
	Papaya ringsplot virus (PRSV)	[32]
	Watermelon mosaic virus (WMV)	[28]
	Zucchini yellow mosaic virus (ZYMV)	[32]
ZYMV	Beet yellows virus (BYV)	[4]
	Cucurbit aphid-borne yellows virus (CABYV)	[6]
	Cucumber green mottle mosaic virus (CGMMV)	[4]
	Cucurbit chlorotic yellow virus (CCYV)	[6]
	Cucumber vein yellowing virus (CVYV)	[2]
	Cucurbit yellow stunting disorder virus (CYSDV)	[33]
	Cucumber mosaic virus (CMV)	[6]
	Papaya ringsplot virus (PRSV)	[26]
	Squash mosaic virus (SqMV)	[25]
	Melon necrotic spot virus (MNSV)	[2]

Watermelon mosaic virus (WMV)	[26]

References list:

1. Moreno, A.B.; López-Moya, J.J. When Viruses Play Team Sports: Mixed Infections in Plants. *Phytopathology* **2020**, *110*, 29–48.
2. Kassem, M.A.; Sempere, R.N.; Juárez, M.; Aranda, M.A.; Truniger, V. Cucurbit aphid-borne yellows virus Is Prevalent in Field-Grown Cucurbit Crops of Southeastern Spain. *Plant Dis.* **2007**, *91*, 232–238.
3. Vidal, A.H.; Sanches, M.M.; Alves-Freitas, D.M.T.; Abreu, E.F.M.; Lacorte, C.; Pinheiro-Lima, B.; Rosa, R.C.C.; Jesus, O.N.; Campos, M.A.; Varsani, A.; et al. First World Report of Cucurbit Aphid-Borne Yellows Virus Infecting Passionfruit. *Plant Dis.* **2018**, *102*, 2665.
4. Bananej, K.; Vahdat, A. Identification, distribution and incidence of viruses in field-grown cucurbit crops of Iran. *Phytopathol. Mediterr.* **2008**, *47*, 247–257.
5. Minicka, J.; Zarzyńska-Nowak, A.; Budzyńska, D.; Borodynko-Filas, N.; Hasiów-Jaroszewska, B. High-throughput sequencing facilitates discovery of new plant viruses in Poland. *Plants* **2020**, *9*, 1–20.
6. Chan, Y.L.; Saidov, N.; Lee, L.M.; Kuo, F.H.; Shih, S.L.; Kenyon, L. Survey of Viruses Infecting Tomato, Cucumber and Mung Bean in Tajikistan. *Horticulturae* **2022**, *8*, 1–9.
7. Xiang, H.Y.; Shang, Q.X.; Han, C.G.; Li, D.W.; Yu, J.L. Complete sequence analysis reveals two distinct poleroviruses infecting cucurbits in China. *Arch. Virol.* **2008**, *153*, 1155–1160.
8. Rabadán, M.P., Juárez, M., Gómez, P. Long-term monitoring of aphid-transmitted viruses in melon and zucchini crops: genetic diversity and population structure of cucurbit aphid-borne yellows virus and watermelon mosaic virus. *Phytopathology.* **2023**.
9. Knierim, D.; Tsai, W.S.; Maiss, E.; Kenyon, L. Molecular diversity of poleroviruses infecting cucurbit crops in four countries reveals the presence of members of six distinct species. *Arch. Virol.* **2014**, *159*, 1459–1465.
10. Damayanti, T.A.; Rahmatilah, M.; Listihani; Hidayat, S.H.; Wiyono, S. Cucurbit aphid-borne yellows virus (CABYV) infecting melon and bitter gourd in Java, Indonesia. *IOP Conf. Ser. Earth Environ. Sci.* **2021**, *948*.
11. Nikolić, D.; Vučurović, A.; Stanković, I.; Radović, N.; Zečević, K.; Bulajić, A.; Krstić, B. Viruses affecting tomato crops in Serbia. *Eur. J. Plant Pathol.* **2018**, *152*, 225–235.
12. Kim Jeong-Soo; Lee Su-Heon; Choi Hong-Soo; Kim Mi Kyeong; Kwak Hae-Ryun; Kim Jung Sun; Nam Moon; Cho Jeom Deog; Cho In-Sook; Choi Gug-Seoun 2007-2011 Characteristics of Plant Virus Infections on Crop Samples Submitted from Agricultural Places. *Res. Plant Dis. 18*, 277–289.
13. Kumari, A.; Kumar, S.; Chauhan, P.S.; Raj, S.K. Elimination of coexisting canna yellow mottle virus, bean yellow mosaic virus and cucumber mosaic virus from Canna generalis cv. black knight through in vitro chemotherapy of rhizome explants. *3 Biotech* **2022**, *12*, 1–10.
14. Arogundade, O.; Balogun, S.O.; Aliyu, T.H. Effects of cowpea mottle virus and cucumber mosaic virus on six soybean (Glycine max L.) cultivars. *Virol. J.* **2009**, *6*, 1–5.
15. Odedara, O.O.; Kumar, P.L. Incidence and diversity of viruses in cowpeas and weeds in the unmanaged farming systems of savanna zones in Nigeria. *Arch. Phytopathol. Plant Prot.* **2017**, *50*, 1–12.
16. González, L.E.; Peiró, R.; Rubio, L.; Galipienso, L. Persistent southern tomato virus (Stv) interacts with cucumber mosaic and/or pepino mosaic virus in mixed-infections modifying plant symptoms, viral titer and small rna accumulation. *Microorganisms* **2021**, *9*.
17. Murphy, J.F.; Bowen, K.L. Synergistic disease in pepper caused by the mixed infection of

Cucumber mosaic virus and Pepper mottle virus. *Phytopathology* **2006**, *96*, 240–247.
18. Da Silva Barbosa, G.; De Araújo Lima, J.A.; De Queiróz, M.A.; De Cássia Souza Dias, R.; Lima, C.S. Identification and effects of mixed infection of potyvirus isolates with cucumber mosaic virus in cucurbits. *Rev. Caatinga* **2016**, *29*, 1028–1035.
19. Arogundade, O.; Balogun, O.S.; Kareem, K.T. Occurrence and distribution of pepper veinal mottle virus and cucumber mosaic virus in pepper in Ibadan, Nigeria. *Virol. J.* **2012**, *9*, 2–5.
20. Herrera-Vásquez, J.A.; Alfaro-Fernández, A.; Córdoba-Sellés, M.C.; Cebrián, M.C.; Font, M.I.; Jordá, C. First Report of Tomato torrado virus Infecting Tomato in Single and Mixed Infections with Cucumber mosaic virus in Panama. *Plant Dis.* **2009**, *93*, 198.
21. Takeshita, M.; Koizumi, E.; Noguchi, M.; Sueda, K.; Shimura, H.; Ishikawa, N.; Matsuura, H.; Ohshima, K.; Natsuaki, T.; Kuwata, S.; et al. Infection dynamics in viral spread and interference under the synergism between Cucumber mosaic virus and Turnip mosaic virus. *Mol. Plant-Microbe Interact.* **2012**, *25*, 18–27.
22. Alabi, O.J.; Al Rwahnih, M.; Jifon, J.L.; Sétamou, M.; Brown, J.K.; Gregg, L.; Park, J.W. A mixed infection of Lettuce chlorosis virus, papaya ringspot virus, and tomato yellow leaf curl virus-IL detected in a Texas papaya orchard affected by a virus-like disease outbreak. *Plant Dis.* **2017**, *101*, 1094–1102.
23. Noa-Carrazana, J.C.; González-de-León, D.; Ruiz-Castro, B.S.; Piñero, D.; Silva-Rosales, L. Distribution of Papaya ringspot virus and Papaya mosaic virus in Papaya Plants (Carica papaya) in Mexico. *Plant Dis.* **2006**, *90*, 1004–1011.
24. Huo, P.; Shen, W.T.; Yan, P.; Tuo, D.C.; Li, X.Y.; Zhou, P. Simultaneous detection of papaya ringspot virus, papaya leaf distortion mosaic virus, and papaya mosaic virus by multiplex real-time reverse transcription PCR. *Acta Virol.* **2015**, *59*, 380–388.
25. Ali, A.; Mohammad, O.; Khattab, A. Distribution of viruses infecting cucurbit crops and isolation of potential new virus-like sequences from weeds in Oklahoma. *Plant Dis.* **2012**, *96*, 243–248.
26. Yeşil, S. Detection of Some Virus Diseases of Edible Seed Squash (Cucurbita pepo L.) in Nevşehir Province, Turkey. *Selcuk J. Agric. Food Sci.* **2020**, *34*, 49–56.
27. Domingo-Calap, M.L.; Moreno, A.B.; Pendón, J.A.D.; Moreno, A.; Fereres, A.; López-Moya, J.J. Assessing the impact on virus transmission and insect vector behavior of a viral mixed infection in melon. *Phytopathology* **2020**, *110*, 174–186.
28. De Moya-Ruiz, C.; Rabadán, P.; Juárez, M.; Gómez, P. Assessment of the current status of potyviruses in watermelon and pumpkin crops in spain: Epidemiological impact of cultivated plants and mixed infections. *Plants* **2021**, *10*, 1–14.
29. Jossey, S.; Babadoost, M. Occurrence and distribution of pumpkin and squash viruses in Illinois. *Plant Dis.* **2008**, *92*, 61–68.
30. Mumo, N.N.; Mamati, G.E.; Ateka, E.M.; Rimberia, F.K.; Asudi, G.O.; Boykin, L.M.; Machuka, E.M.; Njuguna, J.N.; Pelle, R.; Stomeo, F. Metagenomic Analysis of Plant Viruses Associated With Papaya Ringspot Disease in Carica papaya L. in Kenya. *Front. Microbiol.* **2020**, *11*.
31. Read, D.A.; Muoma, J.; Thompson, G.D. Metaviromic analysis reveals coinfection of papaya in western Kenya with a unique strain of Moroccan watermelon mosaic virus and a novel member of the family Alphaflexiviridae. *Arch. Virol.* **2020**, *165*, 1231–1234.
32. Roggero, P.; Gotta, P.; Stravato, V.M.; Dellavalle, G.; Ciuffo, M. Further spread of Moroccan watermelon mosaic potyvirus in Italy in 1998. *J. Plant Pathol.* **1999**, *81*.
33. Gil-Salas, F.M.; Peters, J.; Boonham, N.; Cuadrado, I.M.; Janssen, D. Co-infection with Cucumber vein yellowing virus and Cucurbit yellow stunting disorder virus leading to synergism in cucumber. *Plant Pathol.* **2012**, *61*, 468–478.
